# Working From Home: Experiences of Home-Working, Health Behavior and Well-Being During the 2020 UK COVID-19 Lockdown

**DOI:** 10.1097/JOM.0000000000002757

**Published:** 2022-11-17

**Authors:** Samuel Keightley, Myanna Duncan, Benjamin Gardner

**Affiliations:** From the Department of Psychology, Institute of Psychiatry, Psychology and Neuroscience, King’s College London, De Crespigny Park, London, SE5 8AF, UK (S.K., M.D., B.G.); and Department of Psychology, University of Surrey, Guildford, Surrey, GU2 7XH, UK (B.G.).

**Keywords:** occupational psychology, work practices, health behaviors, health, well-being, Covid-19

## Abstract

The Spring 2020 UK COVID-19 lockdown required normally office-based workers to modify their work-related practices to work at home. This study explored workers' experiences of adapting to home working, health behaviors and well-being.

**Methods:**

Twenty-seven home working employees (19 women; aged 23–57 years), from various industry sectors, gave individual semi-structured interviews. Topics focused on home working experiences, routine adaptations, and changes in health behaviors and well-being.

**Results:**

Four themes were extracted: changes to the work interface; adaptations to a new workspace; changes to work-life balance; and adjustments to a new social context. Notably, participants reported greater reliance on computer-based interactions, which they felt discouraged physical activity and increased sitting. Working in a domestic environment reportedly challenged work-home boundaries.

**Conclusions:**

Work practices can incidentally detrimentally impact health-related behaviors and well-being. Organizations should develop policies and procedures to promote health-conducive home working.

## Abstract 2

This study shows that adaptations to working practices, in response to the Spring 2020 UK Covid-19 lockdown, incidentally affected health behaviours (e.g., physical activity, sedentary behaviour, dietary consumption) and wellbeing. Outside of the pandemic context, this suggests that organisational policies should encourage home-working practices that shield employee health and wellbeing.

**LEARNING OUTCOMES**

1. Adopt a goal theory perspective to describe how work practices may incidentally affect health behavior and well-being
2. Describe normally office-based participants' experiences of adaptations to their working practices, health behavior and well-being during the 2020 Covid-19 lockdown
3. Identify potential strategies for encouraging home-working practices conducive to health-promoting behavior and positive well-being

In March 2020, in response to the COVID-19 pandemic, the UK government imposed a national lockdown that required employees to work from home where possible.^1^ Office-based employees had to adapt their working patterns to home-based working, and many organizations continued to operate near-normally despite this shift to home working. This has led to the adoption of “hybrid” working policies, encouraging employees to alternate between working at the workplace and at home.^2^ Little research has, however, investigated the potential implications of home working for health and well-being.^3,4^ A wealth of research has documented detrimental changes to health behavior during the pandemic, such as decreased physical activity, diet, and sleep quality.^5–7^ Such behavioral changes have typically been attributed to the impacts of self-isolation, shielding, loneliness, confinement, and increased leisure screen time.^5–7^ Little is known about the extent to which shifting to home working during the pandemic may have impacted health behavior.^8^

Previous research centring on the workplace has shown that work-oriented practices and patterns demonstrate the potential to affect health behavior and well-being.^9^ For example, in one study, accelerometer data from UK office workers showed that, on workdays, step count was highest during the morning and evening commute, and at lunch time.^10^ This shows that traveling to the workplace, and taking a lunch break, incurs physical activity. Furthermore, the greatest amount of sitting over the waking day was observed during working hours.^10^ This echoes existing evidence indicating that office-based work typically accumulates prolonged sitting.^11–13^ The physical layout of the workplace environment can influence workers' daily movement and sitting.^9,14^ One study tracked changes in activity among office workers following a move from a “traditional” office environment to a workplace spread across multiple floors with accessible staircases, height-adjustable workstations and standing-permissive meeting rooms.^15^ Device-monitored standing time and step count were found to increase while sitting time decreased.^15^ Studies of dietary behavior among office workers have shown greater unhealthy snacking in workplaces that offer greater proximity to or accessibility of unhealthy snacks, such as on-site shops and vending machines.^16,17^ The social environment of the workplace can also determine health-related behavior. For example, in-person interactions may require walking to colleagues' workspaces or meeting rooms.^18^ Conversely, participants in workplace sitting-reduction interventions often describe social norms around appearing dutiful and productive, which can discourage taking breaks from sitting.^19,20^ Other studies have documented how colleagues create local cultural norms within the office that prescribe unhealthy food choices, such as sharing cakes or biscuits with colleagues or clients.^21,22^ Evidence from dieting workers has shown that colleagues can be a source of support for healthy eating, demonstrating the potential impact of the workplace social environment on eating behaviors.^23^

Theoretically, the relationship between work practices and health behavior can be understood using the COM-B model,^24^ and insights from goal theories.^25^ The COM-B model proposes that all behaviors require capability, opportunity and motivation to be performed.^24^ Goal theories help to clarify the structure of motivation that drives everyday health behaviors in a work setting. Goal theories adopt a hierarchical model of action, whereby outcome goals are situated at higher levels of the hierarchy (e.g., “be productive”), and are served by discrete behaviors at lower levels (e.g., “go to work,” “sit at my desk”^25,26^). Studies demonstrate that, for many workers, work-related productivity is a prioritized goal,^27,28^ pursuit of which can incidentally elicit or inhibit health-related behaviors. For example, the act of commuting to the workplace necessitates physical activity,^10^ and office work undertaken at a desk typically incurs prolonged sitting.^29^ Such behaviors are motivated not by health, but rather by their utility for achieving higher-order work-related goals.^27^ The pursuit of productivity goals also shapes the opportunities available for health-related behaviors, and workers' perceptions of their capability to engage in such behaviors. For example, the greater accessibility of food in the home environment increases opportunities for food consumption, and may diminish workers' perceptions of their capabilities to resist temptations.

Discontinued exposure to the physical and social environment of the workplace during the pandemic likely affected motivation, capability, and opportunity to engage in health behavior, and so health, among home workers. Following the shift to home working during the pandemic, many of the instrumental health-related behaviors centering on the workplace were no longer required to meet work motives. For example, the removal of the daily commute with colleagues rendered incidental physical activity redundant for work purposes, and lessened the opportunity to accrue physical activity during work-related activity. Similarly, the shift to online communication may have increased the importance of sitting at work, diminished opportunities for physical movement, and lessened perceptions of capability to reduce sitting among workers keen to meet perceived norms of being available to colleagues.^30^

Although the impact of home working on health behavior during the pandemic remains underresearched, whether employees work in the workplace or the home environment could have both positive and negative impacts on health behavior and well-being. For example, some commentators have argued that working within the home environment gives workers a greater sense of autonomy^31^ and can reduce work-related distractions.^32^ This may enhance productivity and potentially lower work-related stress. Others, however, have voiced concern about work and leisure boundaries becoming blurred when working from home, leading to extended working hours, disruptions to work-life balance, and greater stress.^33^ Similarly, not having to commute may allow workers greater time to engage in health-promoting behaviors, such as home based physical activity.^34^ Conversely, levels of incidental physical activity will likely decline for many people when commuting is no longer necessary.^10^ Lastly, the discontinued exposure to “office cake” culture offered by home working may minimize some food-based temptations,^35^ but the greater availability of food at home may increase food consumption.^36^

Home-working is expected to become more prevalent after the COVID-19 pandemic ends,^28^ but little is known about how home working may impact health and well-being.^8^ Understanding how home working practices may affect health behaviors and well-being more broadly could provide valuable insights to support health-conducive, postpandemic working practices and policies.^8^ The present study aims to document individuals' experiences of working from home during the first UK COVID-19 pandemic lockdown. Our research question was: “During the pandemic, how did office-based workers experience working from home, health related behaviors, and well-being?”

## METHOD

### Participants and Procedure

Participants were recruited via a study advertisement during April to May 2020 circulated via social media (LinkedIn, Twitter), inviting individuals to take part in an interview exploring their experience of working from home during the COVID-19 pandemic. A £15 shopping voucher was offered as an incentive for completion of the interview. The advertisement contained a URL link where potential participants confirmed their eligibility and provided their email address to allow us to contact them. Eligibility criteria were as follows: UK based adults (18+ years of age); working from home full-time, due to the COVID-19 pandemic; no caring responsibilities for preteen or older adults.

Potential participants were then contacted via email to arrange an interview and were prompted to complete an online survey aimed at ensuring participant eligibility and gathered participant consent and demographic information (age, gender, industry, occupational role). Of 36 individuals who provided their email address, 27 (75%; 19 female, 8 male) took part in semi-structured interviews. Participants were aged 23 to 57 years (M = 29 years, SD = 6.75), and worked in a range of industry sectors, including education (seven), information services (five participants), retail (three), public relations (three), software (two), charity (two), healthcare (two), finance (two), and recruitment (one). Length of employment in current roles ranged between 0 and 13 years.

### Interview Schedule

Semi-structured interviews were conducted by phone or video call (using Skype, Teams, or Zoom) according to participant preference. Interviews were recorded via an external recording device and ranged between 31 and 90 minutes (mean 59 minutes). Interviews covered topics including: experiences of working from home; home working and workplace-based practices; adaptations to home working environments; everyday work and leisure routines; and changes in health behavior, health and well-being. Interviews were conducted by S.K., a male doctoral research student who received formal interview training, while receiving regular supervision from BG. All procedures were approved by the King's College London ethics committee (MRSP-19/20-18230).

### Analysis

Digital interview recordings were automatically transcribed via transcription software Otter.^37^ Transcripts were manually error checked by S.K. Analysis was organized using NVivo 12.^38^ We used Thematic Analysis, underpinned by critical realist assumptions.^39,40^ Our analysis approach, blended elements of “codebook” Thematic Analysis, by inductively generating potential codes and themes early on to aid later coding processes, and “reflexive” Thematic Analysis, whereby analysis remained grounded in the data, and early coding structures were refined in response to insights from later coding.^41^ Our “codebook” methods involved two authors (S.K. and B.G.) independently coded three transcripts and through discussion, agreed on a preliminary thematic “codebook.” This thematic structure was applied and incrementally refined by S.K. to extract, review and name themes from all remaining transcripts. S.K. met regularly with BG, a senior qualitative researcher, throughout the coding process to verify the credibility of analysis.^42^

### Researcher Positionality

The study was conceived jointly by all authors in late March 2020, in response to the first UK COVID-19 lockdown. At that time, S.K. was a first-year doctoral research student, undertaking research to investigate the relationship between home working, health behavior and health among office workers. Before the pandemic, all authors were predominantly office-based and desk-based, working from home at most once weekly. All authors worked from home, due to the pandemic, during the data collection period. Author BG, a social psychologist working mostly in health psychology, has previously published research based on understanding and promoting reductions in sitting and, during the data collection period, took daily walks and attempted daily workouts to maintain health and well-being. MD, an occupational psychologist with expertise in intervention research in work and well-being, was on maternity leave during the study period. S.K. has conducted research investigating the work-related well-being outcomes of office workers experiencing perceived isolation, and during data collection, engaged in regular daily cycling to finish off his working day.

## RESULTS

Four distinct themes were identified, each of which related to a potential aspect of adaptation to home working that potentially affected participants' health behaviors or well-being: changes to the work interface; adaptations to a new workspace; changes to work-life balance; and adjustments to a new social context.

### Changes to the Work Interface

Participants reported a greater dependence on digital technology to work from home, which appeared to reduce incidental physical activity and increase sitting time. The loss of impromptu interactions with co-workers, and an increase in formal work meetings appeared to impact workload, stress, and well-being.

Although impromptu communication remained possible, most participants felt communicating with colleagues from home required greater purpose, organization, and formal scheduling—i.e., a “meeting spot in your diary” (P35)–than when in the workplace. Home-working was felt to preclude the spontaneous and potentially productive in-person communication available in the workplace:

*I feel that things [are] getting done a little bit slower just because you can’t have those quick chats ... that I would usually have in the office. (P26)*

The reduction in informal communication with co-workers was mirrored by a reported increase in daily meetings (*“I probably [now] spend about three hours a day in different calls … which is quite a lot”; P9).* Attending multiple consecutive meetings limited participants' perceived opportunity to take breaks (“*you don’t have a minute or two in between meetings just to go to the bathroom”; P22)* and potentially reduced frequency of eating (“*it’s an ongoing struggle to find the time to eat”; P23).*

Not only were meetings seen as time-consuming and unproductive, but many participants also reported being allocated additional work in meetings. The combination of reduced productivity and increased workload led many to feel obliged to work longer hours:

*When you have five or six hours of calls a day, you know, those all eat into the working time that you have in your normal working day. And often you’re collecting work to do during those calls. [On] a couple of occasions I*'*ve worked later into the evening to just get some stuff done. (P2)*

Many participants also reported using their computers for longer, and remaining within close proximity to their workstation due to a perceived obligation to remain mentally connected to work. This limited their physical movement and so elicited lengthier periods of sitting (“*it’s easy just to end up sitting there for the [whole] day”; P35)*.

Participants reported having to adjust to new behavioral norms in online meetings and calls. When attending large online meetings characterized by one-way flows of information, participants felt more able to attend with their camera off which allowed them to multitask. Some participants used this as an opportunity for physical activity:

*[On] phone calls ... when I am just listening in [...] [I] put my headphones on and then go for the walk. I*'*m still paying attention, but I can still get the walk in. (P23)*

In smaller online meetings however, many felt obligated to not only ensure their video camera was on to demonstrate engagement and conformity with colleagues (*“you get a call that everybody*'*s got their video screen on, so you have to do it too”; P35*), but also to sit (“*[You] have to sit in the same place all the time, especially if [you] have to do calls back to back”; P9)*. Sitting was seen as strongly normative for formal meetings and for hosting meetings.

*If it*'*s just a call, like a discussion or something I can do that kind of stood up or on the sofa, but a lot of what I do is [...] webinars with lots of people coming on to watch me presenting. [...] So I feel like I need to look ... professional rather than just sat on the sofa. So ... I need to sit at my desk and have my two screens and be on webcam and look presentable. (P6)*

### Adaptations to a New Workspace

Participants discussed multiple challenges arising from using the home for work purposes. One challenge was to identify or create physical environments conducive to working. Some had pre-existing areas of the home designed for work activities, such as offices or studies. Others described makeshift workspaces, improvised through transforming or repurposing leisure spaces (“*we bought one of those laptop tables, you can get, so you can sit on the sofa*”; *P19)*. Some reported converting a specific area in the home into a temporary designated workspace at the beginning of each day, which helped differentiate leisure and work time:

*I actually physically move my laptop and notebook and bits and pieces out from underneath where they*'*re sitting under the kitchen table. And then that is kind of, that*'*s the start of the workday. (P23*)

The physical separation of work and leisure was deemed important for achieving a mental separation. Several participants described purposefully removing work-related visual cues to allow them to detach more fully from thoughts about work during leisure time:

*I kind of make sure that my workstation is like pushed into the corner of the room. Because otherwise it feels like you*'*re sat on the sofa watching TV and you*'*ve got work in the corner. (P6*)

Many participants used trial and error to identify an optimal permanent workspace. Attempts were sometimes abandoned, for example because participants found themselves being disturbed by other family members or housemates (*“my mum was coming in and out to make tea ... it was really distracting, so I moved into my room now and that*'*s much better”; P1*). Many participants were unable to find a permanent workspace, so used a variety of spaces. Workspace selection appeared to be driven by the nature of expected work tasks, workload, perceived engagement requirements, and formality:

*If I have a lighter day like today, I am in my bedroom, and work from bed if I just have one or two calls, or [if] they*'*re a bit more casual or informal. (P2)*

Many reported that their home environment was ill-suited to work. For some, the physical environment was deemed incompatible with prolonged computer-based work (“*the table is not really a desk [and] the chairs are not really meant for people to be sitting on them [for long periods]”; P22)*. Several participants described physical comfort and posture problems that they attributed to using household furniture for normally-desk-based work:

*The chair is quite uncomfortable to sit on and work and I don*'*t think it*'*s at the right height and sometimes I*'*m sat on the sofa with my laptop, but then that makes you really achy in the shoulders and neck. (P29)*

For some participants, relative to the workplace context, the home working environment offered more access to food and so convenience, which resulted in greater temptations to snack:

*I feel a bit more of a desire to snack during the afternoons [at home] ... because [food is] within walking distance, and [it*'*s] a little bit harder to kind of control a craving, or you feel like you need something else to eat. Whereas in the office ... we don*'*t really have anywhere we can buy things. (P21)*

The proximity of snacks was especially perceived as an unwanted distraction by those working near or in kitchen spaces. Conversely however, home working discontinued exposure to office snacking culture, so removed some temptations:

*Sometimes people would bring snacks into the office … [like] cakes to eat. There*'*s less [of] that [when working at home]. (P17*)

Participants reported that amenities were in closer proximity, so they tended to accrue minimal incidental physical activity in the course of the workday:

*The toilet [at my workplace] is a few hundred metres away … I*'*m getting a few hundred steps in just going to the toilet. Whereas obviously [at home], the bathroom*'*s right next to my bedroom. (P21)*

Consequently, while some participants reported feeling motivated to take regular activity breaks from sitting, they often found doing so unsatisfactory due to the negligible movement involved (*“I*'*m getting up quite a bit but not really having anywhere to go”; P11*). Some participants adapted to this by taking breaks to undertake household chores and other non–work-related activities. For some, these breaks were designed to reduce sitting time (*“I*'*m always like running around, like washing the dishes—whenever I can get a chance to leave that chair. I do it.”; P22*), whereas others were taken to obtain “thinking space” needed to solve work problems:

*If I*'*ve got some [mental] block ... then I*'*ll just take the dog [for a walk] ... 15 minutes, 20 minutes just to go around the block. And try and calm my mind. Give me some reflection time. (P14*)

### Changes to Work-Life Balance

Participants described various benefits and challenges to work-life balance arising from working at home. The time savings from not commuting, and the flexibility of working at home, made some feel more able to engage in activities potentially beneficial to health and well-being. Some participants invested time savings in sleeping longer, which reportedly enhanced sleep quality (*“I*'*m sleeping better now, and having that time back from commuting has really helped”; P9*), and some reported going to bed later. Others reported spending more time preparing fresh food or diversifying their diet (“*[I*'*ve been able to] do a wider variety of meals because you*'*ve got that bit of extra time from not commuting;” P19*). Not having to travel was felt by some to conserve their physical energy, so allowed them to take more physical activity:

*In a normal situation, you come home from the train from work, you're so tired, you still have to get changed and run to the gym. It demotivates you a little bit sometimes. [But] right now I feel a little bit more motivated. (P5*)

Not having to worry about getting to work on time also reduced stress for some (*“Now I know that my “office” is next door, I don’t feel as anxious, and ... the quality of my sleep is probably better”; P36*).

However, many participants reported that aspects of home working compromised their work-life balance, by blurring the physical and psychological boundaries of work and leisure. Some reported challenges in adhering to normal working hours (*“I’m trying to ... get my job done in my working hours, rather than letting it creep and bleed into personal time.”; P2*). Many described difficulties with “switching off” at the end of the workday, because they remained in close physical—and so psychological—proximity to work tasks and tools:

*It takes me longer to get to sleep now because my mind’s just wearing. ... [I] can’t switch off from work because work is just there all the time. (P6*)

Some participants experienced their daily commute as a valued psychological tool for demarcating work and leisure mindsets:

*Previously, I’ve used the journey home as my transition period from work to family. [I] try not to think about family when I’m at work, [and] try [not] to think about work when I’m at home. However, now work is at home all the time. Sometimes when I wake up in the morning I wake up thinking about work. (P14)*

Some participants sought to replicate this demarcation by engaging in purposeful events to bookend the workday. This event often mimicked the daily commute in that it involved physical activity, such as walking or cycling. Many reported this vitalized them before work, or helped them disconnect and unwind after work:

*I used to walk to work. [...] [Now] I get up and walk at about 7.30 in the morning, and then I get back I’ll have a cup of tea with my parents and boot up my computer. (P30*)

Removing oneself from the home environment (e.g., to go for a walk) appeared valuable for separating work and leisure. Those with less opportunity to move away from the home working environment expressed difficulties in mentally disconnecting:

*I felt like I did everything in the living rooms. [I was] working [in there], and then I used to relax in the living room and then workout. So for me, it was quite hard to kind of detach yourself from work ... Having to do everything in one space is quite difficult. (P36)*

Other participants sought to separate work and non-work time by consuming alcohol, a behavior that for some symbolized leisure:

*I have a glass of wine like every night now. It’s part of that wind-down for me. I know that I shouldn’t be thinking about work when I have my glass of wine. (P6)*

### Adjustments to a New Social Context

Participants experienced notable changes to their immediate social environment, arising both from working in the informal social context of the home and the absence of social influences from the workplace.

Outside of online meetings, participants reported feeling less social pressure from colleagues, due to reduced visibility. This allowed them a newfound freedom to take impromptu breaks from sitting (*“it’s not as if I’m sitting among people [where] everyone else is working so you just work. At home, I feel like, Okay, [I can] just take a break”; P26*). Some participants reported being more productive because they were less likely to be distracted by colleagues (*“even though it’s nice that someone will say ‘[do] you wanna have a coffee’, I just feel obliged to get up even if you’re in the middle of something”; P25*). Conversely, others felt that not being in the physical presence of colleagues was detrimental to their productivity, because they were less accountable and so more susceptible to self-generated distractions:

*There’s no fear of work colleagues looking over my shoulder not seeing that I’m not working. I think it’s a bit dangerous, I find it more difficult to stay focused. (P21)*

The physical absence of co-workers led some to worry that colleagues would suspect they were not working (“*there’s always that fear, you know, that people [will think you’re slacking]”; P14)*. This prompted many to feel obliged to demonstrate that they were ‘getting work done’ (“*it’s kind of got into a culture now, it’s like you’re proving how much work you’ve done in that day”; P6)*, which in turn led participants to feel compelled to remain digitally present, and so seated at their workstation for longer hours:

However, others reported that home working freed them from presenteeism and allowed them the agency to manage their working hours, take more breaks, and achieve a better work-life balance:

*I find it easier to actually only work my hours [at home]. In the office, there is a culture of staying late or working through your lunch break, which I think is bad. (P9*)

Social interaction during the workday was highly valued by many participants, and some reported compensating for the absence of colleagues by replicating their usual sociable workplace-based actions in the home environment. For example, some found their break-taking synchronized with other household members (*“if [my partner] gets up to ... make his lunch or a cup of coffee, naturally I find myself wanting to get up as well ... I’m like that in the office”; P19*). Others reported consciously working in areas of their home conducive to social interaction with household members:

*I just think it’s just nice to sometimes just work in the kitchen and ... if you’re not too busy ... if someone can distract you and you have a bit of a chat. It’s almost like being in a common space where someone can talk to you. (P25*)

Other participants felt restricted by the presence of non-working household members, and sought to avoid them by confining themselves to specific areas of the home during the working day.

*I can’t really leave this room because If do then my parents are in the next room, watching TV or doing everything, they don’t want to disturb me and I don’t want to disturb them. (P30)*

## DISCUSSION

Interviews with normally office-based workers who were required to work exclusively from home during the first UK COVID-19 lockdown documented reported changes to working practices and environments. These changes reportedly had consequences for workers’ health behavior and well-being. Home-working benefitted health behavior and well-being for some, who for example felt more able to control their diet and take breaks from sitting in the physical absence of colleagues, and some reported investing the time saved by not having to commute in health-promoting behaviors like cooking nutritious meals. There were however also reportedly deleterious impacts of home working. For example, some felt that a shift toward digital communication had increased their sitting time and inhibited physical activity; a blurring of physical and psychological boundaries between work and leisure time reportedly compromised well-being; and changes to the accessibility of foods apparently affected dietary consumption. Although home working experiences during the COVID-19 lockdown may not accurately represent more typical home working, our findings have important implications for organizational policy and practices outside of pandemic settings. Organizations should encourage work-related practices that support health and well-being among home workers.

Our research suggests that adaptations to working practices when working at home rather than in the workplace may have incidentally impacted health behaviors and well-being during the COVID-19 lockdown of Spring 2020. For some, not being at the workplace provided valuable opportunities to engage in behaviors conducive to health and well-being. For example, in the physical absence of colleagues, and so social pressure to appear productive, some felt more capable of taking regular breaks from sitting. Some valued the removal of the daily commute because they used the time savings to engage in exercise, to cook meals, or to sleep. For others, however, working from home detrimentally impacted health behavior and well-being. For example, it is well-documented that physical activity decreased and sedentary behaviors increased among home workers during the pandemic.^43–45^ Our participants reported that the discontinuation of in-person communication during the lockdown rendered day to day working practices entirely dependent on digital interfaces (see too^46^). Home-workers experienced a greater number of online meetings, and many described feeling pressured to be visibly online throughout the day to demonstrate to colleagues that they were working. Consequently, many described sitting for long periods and decreases in daily physical activity. Significantly, our participants felt that having to be available online limited their opportunity to take breaks from their workstation. This may explain findings by Spence et al,^47^ who found that a perceived lack of physical opportunity predicted decreased physical activity in a UK sample in early June 2020, during the same COVID-19 lockdown during which we undertook our study. Organizations should actively champion taking activity breaks or incorporating movement into everyday work activities when working from home, as well as communicate clear work time expectations, to prevent digital presenteeism.^48^ One promising way to do this is by encouraging workers to take walks during online meetings, which some of our participants reported doing. While workers and managers often find non-seated meetings inappropriate for formal meetings or group discussions,^20^ going for a walk may be feasible during informal meetings, or those that require minimal input from attendees.

Maintaining work-life boundaries is thought to be crucial for health and well-being, because failing to “switch off” from work can impair work-life balance and lead to exhaustion.^49,50^ Our participants' working routines appeared to be important in shaping work-life boundaries. Although some participants had a dedicated room in which to work, most reported difficulties finding feasible workspaces in their homes, and instead repurposed domestic spaces, such as kitchen counters and dining tables. Such improvisation of workspaces in the home was commonplace during the 2020 COVID-19 lockdown.^51^ Significantly, some of our participants reported feeling unable to disengage from work, because of the continued presence of work cues. This attests to the potential for physical workspace decisions to lead to blurred work-life boundaries. Other participants appeared to maintain boundaries more effectively by purposefully clearing their workspaces when completing their workday. Similarly, the removal of the need to commute appeared consequential for boundary management. Some participants reported finding commuting useful for transitioning between work and home “mindsets,” and so felt less able to detach from work in the absence of the commute. Research shows that transitioning between physical environments can promote psychological detachment.^52^ Some participants appeared mindful of the value of this physical transition, so sought to engage in purposeful activities to start or finish their working time. These not only reportedly helped demarcate work and leisure time but also, because they typically occurred outside of the home, often involved incidental physical activity (e.g., going for a walk). Given that home working typically reduces the physical activity incurred by commuting,^10^ engaging in such boundary management activities may have particular health behavior benefits. Organizations should encourage home workers to replicate the psychological adjustment and health behavior benefits of commuting,^53^ by bookending their work time with non-work events that ideally involve leaving the home and engaging in some form of physical activity, such as walking, running or cycling. We also found that some participants consumed alcohol as a way of “switching off” from work demands. This demonstrates the potential for health-risk behaviors to be adopted for boundary management purposes, which may explain previously established links between work-life conflict and alcohol consumption.^54^ Our study points to the importance of encouraging workers to engage in positive health behaviors to manage work-life boundaries.

Our results suggest that social and physical environment differences between the workplace and the home may influence dietary intake. For example, the physical absence of colleagues reportedly led to a reduction in snacking due to discontinued exposure to snacking norms, such as “cake culture” among co-workers.^21,22^ Conversely however, participants described having greater access to food at home which, whilst appearing to enhance lunch time dietary intake for some, also increased unhealthy snacking. Proximity and perceived availability of food has been shown to impact dietary consumption and health.^55^ For many workers, working at home may increase access to potentially unhealthy foods. Physical environment changes may also affect workers' health behaviors via habit mechanisms. Habitual behaviors are built on learned associations between environments and actions.^56^ For many people, the home environment is likely associated with leisure time behaviors, such as snacking.^57^ Everyday settings and cues within the home (e.g., the kitchen) may therefore act as unconscious triggers to such behaviors in a way that the workplace environment does not. Developing interventions to support healthy eating patterns among home workers may require further research to understand which microenvironments within the home are least conducive to unhealthy dietary consumption. Workers should also be given self-regulatory advice on how to overcome unwanted unhealthy eating habits in the home environment.^58^

From a theoretical perspective, by documenting the impact of changes in everyday work activities on health behavior, our findings demonstrate the importance for health of work-related motives, and the constraints that pursuing work goals imposes on perceived capabilities and opportunities for engaging in health conducive behaviors.^24^ Many work-related activities incidentally facilitate or inhibit health-related behaviors, because such behaviors are congruent or incongruent with work goals. For example, moving around large workspaces involves physical activity,^15^ and is instrumental to interacting with colleagues or using essential facilities. Conversely, using a computer at a desk prolongs sitting and inhibits movement,^29^ but is often necessary to complete work tasks. Our results show how, when working from home, the instrumentality of health-related behaviors to the pursuit of work goals changed significantly. For example, greater use of digital communication, and a smaller physical environment, reduced the importance of movement for interaction when working at home. Indeed, our participants reported that physical activity became largely incongruent with work goals, such that taking activity breaks was seen as potentially compromising their engagement with work. Although some participants made purposeful attempts to engage in health behaviors during or outside of work time, many participants reported engaging in less physical activity and sitting for longer periods than usual. Organizations should support health behavior among home workers by portraying health-promoting activities as instrumental to meeting organizational goals. For example, workers might be encouraged to take walks to obtain “thinking space” when facing difficult tasks, to boost productivity.^59,60^ Encouraging health behaviors as part of work-related activity would likely target all components of the COM-B model.^24^ If behaviors were explicitly encouraged as beneficial for – or, at least, no hindrance to – work purposes, workers should be more motivated, and perceive greater opportunity and capability to engage in them, due to allayed concerns about appearing to be compromising productivity in the pursuit of personal health goals.

Study limitations must be acknowledged. We collected data on home working during the first UK COVID-19 lockdown which required workers and organizations to cease all non-essential workplace-based activity. Organizational policy and practice has evolved since the first lockdown, with many organizations embracing “hybrid” home- and workplace-based working patterns.^2^ Consequently, our participants' experiences could be argued to have limited application to post-pandemic settings. However, many of the phenomena we observed, such as difficulties in managing work-life boundaries when in a domestic setting, are likely to remain relevant beyond the pandemic. We recruited participants from social media platforms, who are likely to be technology literate.^61^ Our sampling method may therefore have precluded experiences among less technologically skilled office workers, for whom greater digital dependence may be a source of anxiety.^62^ In an attempt to restrict our findings to changes in work-related practices rather than changes in experiences of caring for others during lockdown, we excluded workers with dependants. Yet, one in seven workers are thought to have caring responsibilities.^63^ More research is needed to examine the health and well-being impacts of home working for workers with dependants. We did not assess participants' home-working experience before the pandemic. Greater home working experience has been shown to foster development of more effective work-life balance management strategies.^3^ Our participants' experiences may reflect a relative lack of experience of adaptation to home working, which may have dissipated over the longer term as they became accustomed to home -working and spontaneously adopted health-protective strategies.^3^ In addition, we did not consider individual differences in our study. Individual differences, such as how people respond to work pressure, may have shaped how our participants responded to changes in home working practices during the pandemic.^64^ For example, employees scoring higher on neuroticism tend to experience greater problems managing work-life balance.^65^ Future research should examine the extent to which individual differences influence how changes in work practices affect health and well-being.

Although we attempted to capture all relevant health behaviors via the interviews, it is possible that our prior research interest in sedentary behavior may have influenced interview and analysis procedures, such that participants' accounts emphasized sitting and physical activity more than other health behaviors that may have changed in line with home working practices.^66^ Lastly, the qualitative nature of our study precludes estimation of the magnitude of impact on health and well-being of each of the work practice changes we documented. Future research is needed to quantify the effects of home working practices on worker health and well-being.

Previous studies have identified significant detriments to health behavior, health and well-being during COVID-19 pandemic lockdowns.^5–7^ Our study suggests that some of these detriments may have arisen from changes to work practices that occurred when office workers were instructed to work from home, rather than their usual workplace. Our findings illustrate the importance of recognizing, outside of pandemic settings, that home working requires adaptations to workplace-based practices, and that these adaptations can potentially affect workers' health and well-being. We encourage organizations to champion policies and practices that support employees' health and well-being when working from home.
